# High glycolytic activity of tumor cells leads to underestimation of electron transport system capacity when mitochondrial ATP synthase is inhibited

**DOI:** 10.1038/s41598-018-35679-8

**Published:** 2018-11-26

**Authors:** Juliana S. Ruas, Edilene S. Siqueira-Santos, Erika Rodrigues-Silva, Roger F. Castilho

**Affiliations:** Department of Clinical Pathology, Faculty of Medical Sciences, University of Campinas (UNICAMP), Campinas, SP Brazil

## Abstract

This study sought to elucidate how oligomycin, an ATP synthase blocker, leads to underestimation of maximal oxygen consumption rate (_max_OCR) and spare respiratory capacity (SRC) in tumor cells. T98G and U-87MG glioma cells were titrated with the protonophore CCCP to induce _max_OCR. The presence of oligomycin (0.3–3.0 µg/mL) led to underestimation of _max_OCR and a consequent decrease in SRC values of between 25% and 40% in medium containing 5.5 or 11 mM glucose. The inhibitory effect of oligomycin on CCCP-induced _max_OCR did not occur when glutamine was the metabolic substrate or when the glycolytic inhibitor 2-deoxyglucose was present. ATP levels were reduced and ADP/ATP ratios increased in cells treated with CCCP, but these changes were minimized when oligomycin was used to inhibit reverse activity of ATP synthase. Exposing digitonin-permeabilized cells to exogenous ATP, but not ADP, resulted in partial inhibition of CCCP-induced _max_OCR. We conclude that underestimation of _max_OCR and SRC in tumor cells when ATP synthase is inhibited is associated with high glycolytic activity and that the glycolytic ATP yield may have an inhibitory effect on the metabolism of respiratory substrates and cytochrome *c* oxidase activity. Under CCCP-induced _max_OCR, oligomycin preserves intracellular ATP by inhibiting ATP synthase reverse activity.

## Introduction

Mitochondrial oxidative metabolism has received increasing attention in different areas of cell biology research, including cell survival, growth and differentiation^1–3^. Some features of oxidative metabolism in tumor cells were characterized several decades ago, and two well-known metabolic properties, the Crabtree and Warburg effects, were described. The former involves the glycolytic metabolism-induced inhibition of mitochondrial oxidative phosphorylation^4,5^, and the latter involves a high glycolytic metabolism that results in the partial oxidation of glucose to pyruvate and its conversion to lactate even in the presence of molecular oxygen^6,7^.

Recently, there has been increased interest in the analysis of mitochondrial-function parameters in tumor cells, and this has been reflected in the growing number of studies showing the importance of mitochondrial oxidative metabolism in tumor cell pathophysiology^3,8,9^. One of the most common approaches used to evaluate mitochondrial bioenergetics in intact cells is measurement of the cellular oxygen consumption rate (OCR)^10–12^. The development of more accurate and user-friendly equipment for measuring oxygen consumption by intact or plasma membrane-permeabilized cells has contributed decisively to this field^10,11,13^. Measurements such as basal cellular respiration, maximal OCR (_max_OCR), spare respiratory capacity (SRC) (i.e., the difference between _max_OCR and basal respiration), the fraction of oxygen consumption related to ATP regeneration and other parameters can be assessed using standard experimental protocols^10–14^.

The maximal capacity of the electron transport system (ETS) can be estimated by promoting protonophore-induced _max_OCR. To determine an additional parameter in the same experimental run, the ATP synthase inhibitor oligomycin is usually added before the protonophore, and the fraction of basal OCR related to ATP regeneration is obtained. However, the presence of oligomycin leads to significant inhibition of _max_OCR, resulting in underestimation of SRC in tumor cell lines^14^. We therefore recently proposed that _max_OCR and SRC in tumor cells should preferably be estimated in the absence of oligomycin^14^.

The present study aimed to further characterize and identify the mechanisms that lead to the underestimation of _max_OCR and SRC in tumor cells when ATP synthase is inhibited. The results indicate that the inhibitory effect of ATP synthase blockers on _max_OCR induced by the protonophore CCCP in tumor cells is associated with high glycolytic activity and maintenance of intracellular ATP levels.

## Results

### Occurrence of oligomycin-induced underestimation of _max_OCR and SRC in T98G glioma cells under different experimental conditions

**Figure 1:**
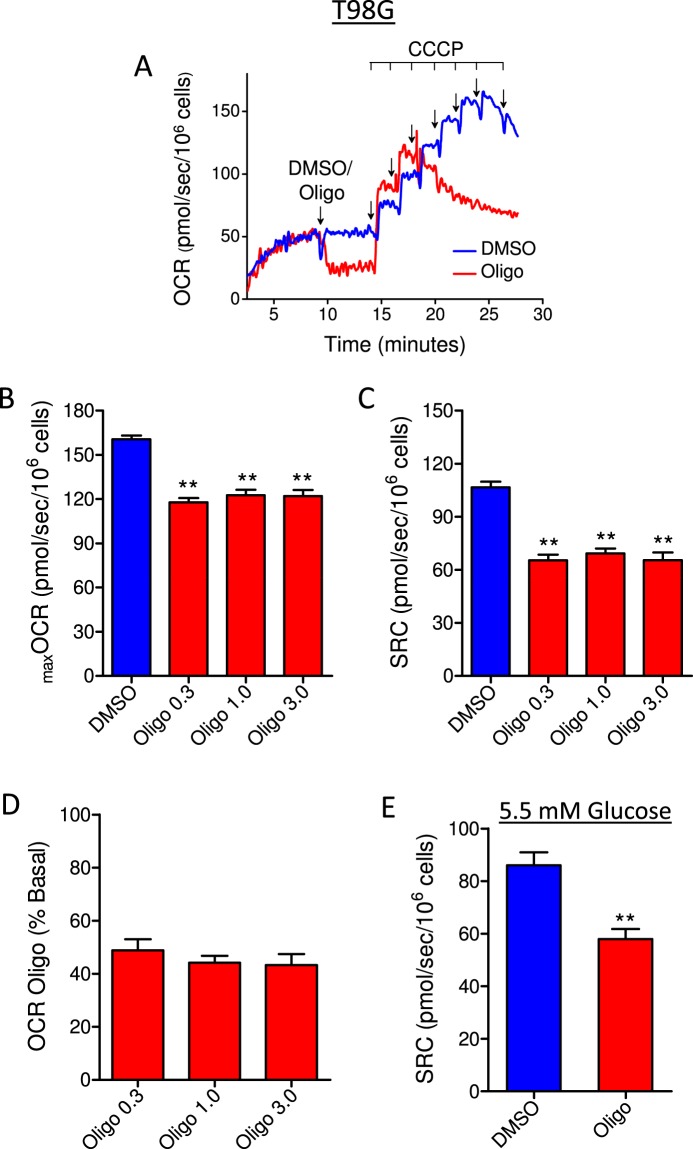
Effect of different concentrations of oligomycin and glucose on CCCP-induced maximal oxygen consumption rate (_max_OCR) in T98G human glioma cells. T98G cells (1 × 10^6^/mL) were resuspended in sDMEM containing 20 mM HEPES and 11 mM (**A–D**) or 5.5 mM (**E**) glucose. CCCP-induced _max_OCR was determined with different concentrations of oligomycin (0.3, 1.0 and 3.0 µg/mL) or without oligomycin, which was replaced by an equal volume of DMSO (0.5 µL). (**A)** Representative traces of OCR in T98G cells incubated in sDMEM. Where indicated by arrows, DMSO (0.5 µL) or 1 µg/mL oligomycin (Oligo) was added, followed by sequential additions of CCCP (2 µM each). (**B**,**C)** Effect of different concentrations of oligomycin on _max_OCR and SRC (i.e., the difference between _max_OCR and basal OCR). Statistically significant difference in relation to the control (DMSO), **P < 0.01. (**D**) Effect of different concentrations of oligomycin on basal OCR. The data are expressed as a percentage of basal OCR. (**E)** Effect of oligomycin (1.0 µg/mL) on SRC values for T98G cells incubated in sDMEM containing 5.5 mM glucose. Statistically significant difference in relation to the control (DMSO), **P < 0.01.

The concentration of oligomycin normally used in experimental protocols is 1 µg/mL, while the minimal concentration to inhibit ATP synthase completely in intact tumor cells is approximately 0.1 µg/mL^14,15^. A wide range of oligomycin concentrations (0.3, 1.0 and 3.0 µg/mL) was tested on OCR parameters in T98G cells. Similar underestimation of CCCP-induced _max_OCR and SRC was observed with the oligomycin concentrations tested (Fig. 1A–C). Figure 1D shows that the different oligomycin concentrations induced similar inhibitory effects on basal OCR, reflecting the fraction of oxygen consumption related to ATP synthesis and indicating that the oligomycin concentrations tested were equally efficient at inhibiting ATP synthase. Figure 1E depicts the effect of oligomycin on SRC when T98G glioma cells were incubated at a glucose concentration found under normoglycemic conditions (i.e., 5.5 mM), instead of 11 mM. Under this condition, the SRC value was 31.6 ± 4.2% lower when estimated in the presence of oligomycin than in the control (vehicle (DMSO) without oligomycin).

**Figure 2:**
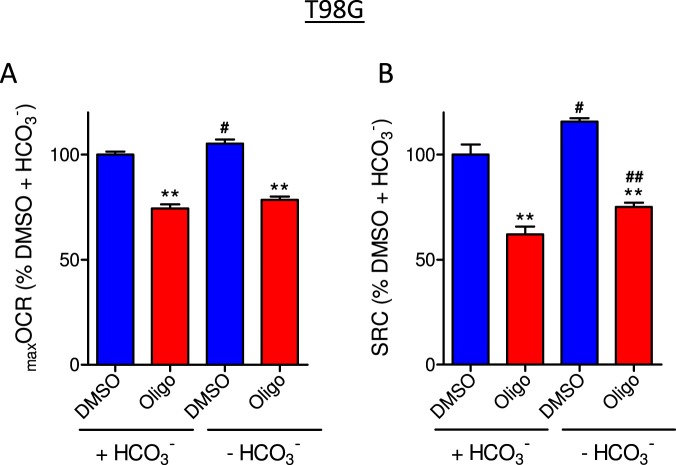
Effect of sodium bicarbonate on CCCP-induced _max_OCR in T98G glioma cells. T98G cells (1.5 × 10^6^/mL) were incubated in sDMEM containing 20 mM HEPES with or without 44 mM sodium bicarbonate (HCO_3_^−^). The experiments were conducted by adding DMSO (0.5 µL) or 1 µg/mL oligomycin (Oligo) after basal respiration was reached, followed by sequential additions of CCCP (2 µM each). (**A**,**B**) Effect of oligomycin on CCCP-induced _max_OCR and estimated SRC for T98G cells. The data were normalized as a percentage of _max_OCR (**A**) or SRC (**B**) observed under the DMSO condition in the presence of HCO_3_^−^ (% DMSO + HCO_3_^−^). Statistically significant difference in relation to the control (DMSO), **P < 0.01. Statistically significant difference in relation to the corresponding condition with sodium bicarbonate, ^#^*P* < 0.05 and ^##^*P* < 0.01.

**Figure 3:**
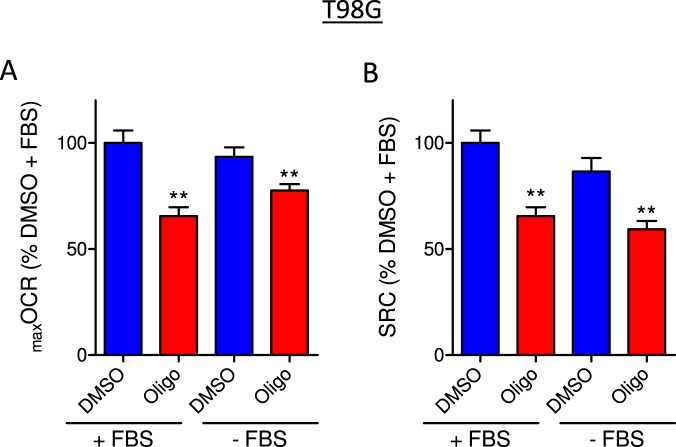
Effect of medium supplementation with fetal bovine serum on CCCP-induced _max_OCR in T98G glioma cells. T98G cells (1.0 × 10^6^/mL) were incubated in DMEM containing 20 mM HEPES with or without 10% fetal bovine serum (FBS). The experiments were conducted by adding DMSO (0.5 µL) or 1 µg/mL oligomycin (Oligo) after stable basal respiration rate was reached, followed by sequential additions of CCCP (+FBS: 2 µM in each addition, −FBS: 0.3 µM in each addition). (**A**,**B**) Effect of oligomycin on CCCP-induced _max_OCR and estimated SRC for T98G cells. The data were normalized as a percentage of _max_OCR (**A**) or SRC (**B**) observed under the DMSO condition in the presence of FBS (% DMSO + FBS). Statistically significant difference in relation to the control (DMSO), **P < 0.01.

The influence of sodium bicarbonate (Fig. 2) and FBS (Fig. 3) on the oligomycin-induced underestimation of _max_OCR and SRC was also tested in T98G cells. Figure 2 shows the _max_OCR and SRC for intact T98G cells incubated in the presence or absence of sodium bicarbonate. Increased _max_OCR and SRC (Fig. 2) was observed in the absence of sodium bicarbonate under control conditions (DMSO). Nevertheless, similar oligomycin-induced underestimation of _max_OCR and SRC was observed both in the presence and absence of sodium bicarbonate. The oligomycin-induced inhibition of SRC in the presence and absence of sodium bicarbonate was 38.0 ± 2.5% and 35.2 ± 1.5%, respectively.

Next, the effect of FBS was evaluated on the oligomycin-induced inhibition of _max_OCR and SRC in T98G cells (Fig. 3). As expected, a lower concentration of CCCP (not shown) was required to achieve _max_OCR in the absence of FBS as this protonophore can bind non-specifically to FBS proteins. We observed a non-significant trend toward lower _max_OCR and SRC in the absence of FBS. Oligomycin-induced underestimation of _max_OCR was 19.9 ± 3.9% in the presence of FBS and 15.9 ± 2.2% in its absence, whereas underestimation of SRC was 33.7 ± 4.3% in the presence of FBS and 30.0 ± 2.4% in its absence.

**Figure 4:**
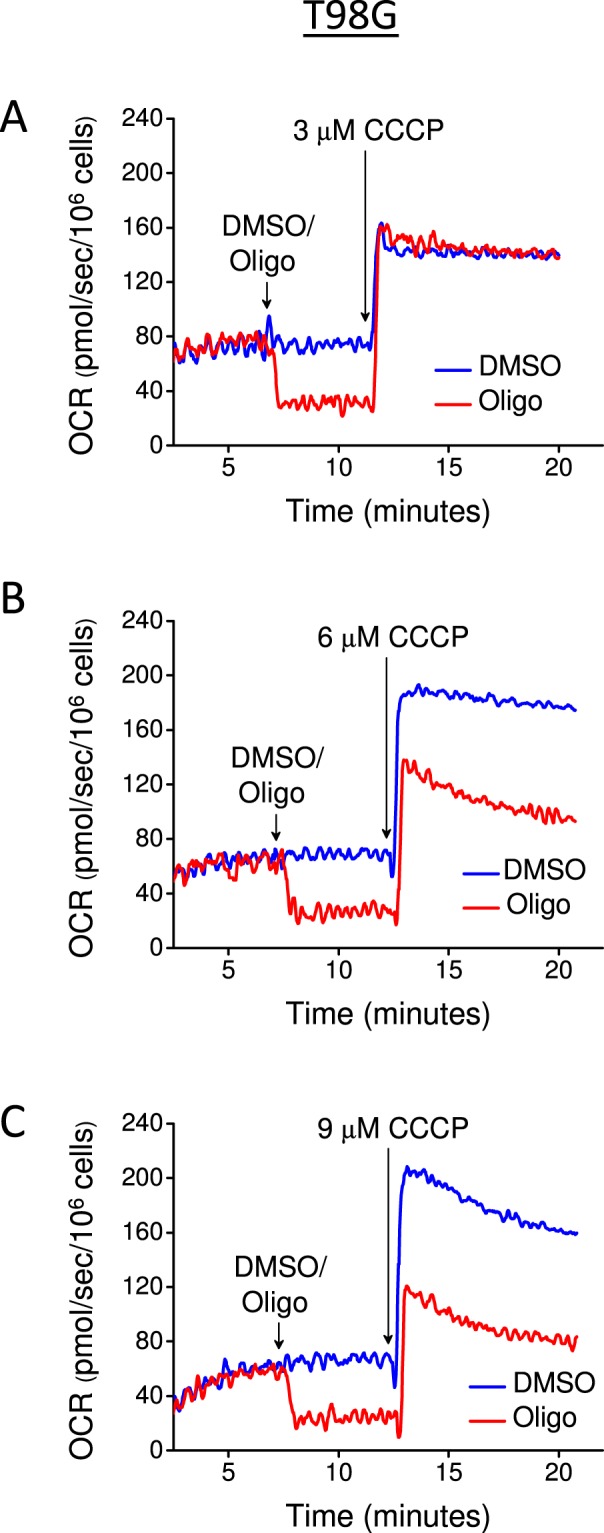
Inhibitory effect of oligomycin on _max_OCR induced by a single addition of CCCP to T98G human glioma cells. T98G cells (1.5 × 10^6^/mL) were incubated in sDMEM containing 20 mM HEPES. Where indicated by the arrows, 0.5 μL DMSO or 1 μg/mL oligomycin (Oligo) was added to the cells, followed by a single addition of CCCP (**A:** 3 μM; **B:** 6 μM; **C:** 9 μM).

The possible interference of multiple CCCP additions with a consequent long exposure time to oligomycin and CCCP to assess _max_OCR and SRC in cell lines was investigated by performing a single addition of CCCP to T98G cells (Fig. 4). First, a suboptimal CCCP concentration (3 µM) was added, and this resulted in similar stimulation of OCR in the absence or presence of oligomycin (Fig. 4A). A 6 µM CCCP concentration was then tested, and a higher OCR was observed under the control condition (DMSO) but lower stimulation of OCR was observed when oligomycin was present (Fig. 4B). Finally, _max_OCR was achieved under DMSO conditions using 9 µM CCCP, although progressive inhibition of _max_OCR occurred immediately after addition of 9 µM CCCP under both the control and oligomycin conditions (Fig. 4C).

Taken together, these results indicate that the inhibitory effect of oligomycin on _max_OCR and, consequently, SRC is related neither to the characteristics of the medium (i.e., glucose concentration and the presence or absence of bicarbonate buffer and FBS) nor to excess concentrations of or long exposure to oligomycin or CCCP.

### The high glycolytic activity of tumor cells leads to underestimation of _max_OCR and SRC in the presence of oligomycin

**Figure 5:**
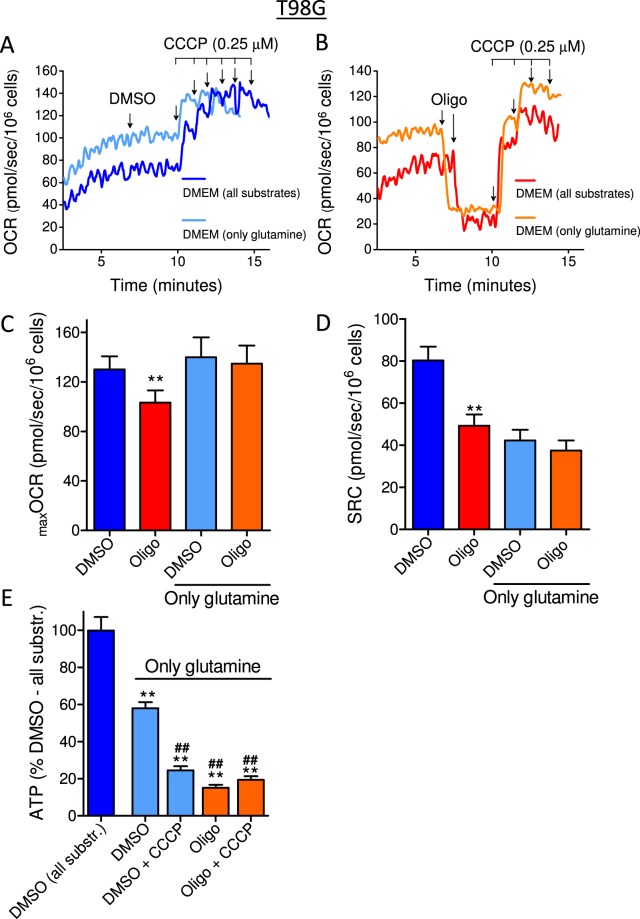
Effect of oligomycin on CCCP-induced _max_OCR in T98G glioma cells incubated in medium containing glutamine as the only metabolic energy substrate. T98G cells (1.5 × 10^6^/mL) were incubated in non-supplemented DMEM containing 20 mM HEPES and all the metabolic energy substrates (11 mM glucose, 4 mM glutamine and 1.25 mM pyruvate) or only 4 mM glutamine. (**A**,**B**) Representative traces of OCR in T98G cells incubated in medium containing all the metabolic energy substrates or only glutamine. Where indicated by arrows, DMSO (0.5 µL) or 1 µg/mL oligomycin (Oligo) was added, followed by sequential additions of CCCP (0.25 µM each). (**C**,**D**) Effect of oligomycin on CCCP-induced _max_OCR (**C**) and estimated SRC (**D**) for T98G cells. Statistically significant difference in relation to the control (DMSO), **P < 0.01. (**E**) Measurements of ATP levels in T98G cells incubated in medium containing all the metabolic energy substrates or only glutamine. Data were normalized as a percentage of the values of ATP in DMSO and all the substrates (% DMSO – all substr.). Statistically significant difference in relation to the control (DMSO) containing all substrates, **P < 0.01. Statistically significant difference in relation to the control (DMSO) containing only glutamine, ^##^*P* < 0.01.

**Figure 6:**
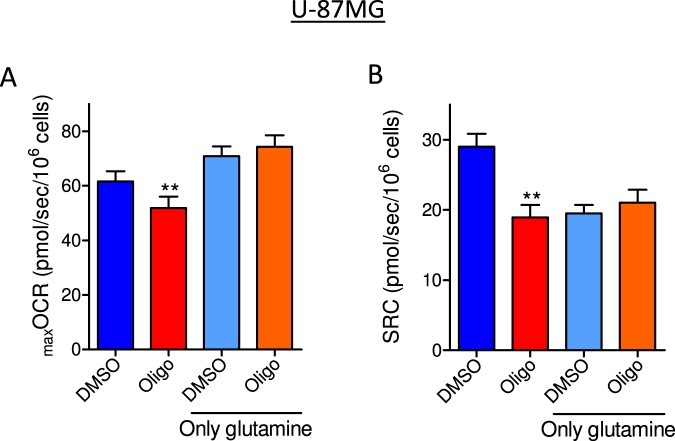
Effect of oligomycin on CCCP-induced _max_OCR in U-87MG glioma cells incubated in medium containing glutamine as the only metabolic energy substrate. U-87MG (2 × 10^6^/mL) cells were incubated in non-supplemented DMEM containing 20 mM HEPES and all the metabolic energy substrates (11 mM glucose, 4 mM glutamine, 1.25 mM pyruvate) or only 4 mM glutamine. (**A**,**B)** Effect of oligomycin on CCCP-induced _max_OCR (**A**) and estimated SRC (**B**) for U-87MG cells. Statistically significant difference in relation to the control (DMSO), **P < 0.01.

**Figure 7:**
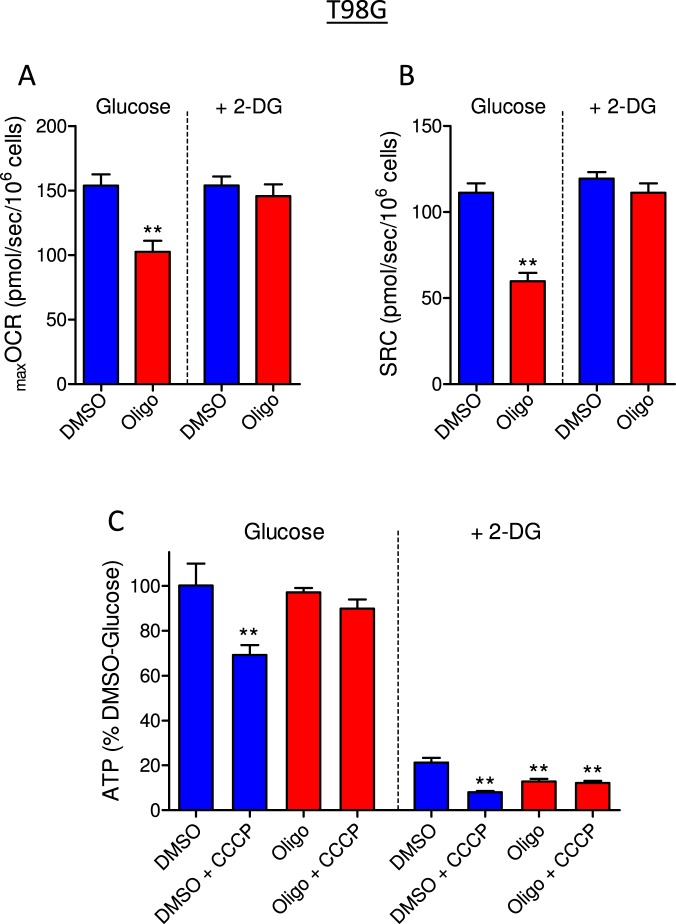
Effect of 2-deoxyglucose (2-DG) on oligomycin-induced underestimation of _max_OCR and on ATP levels in T98G glioma cells. T98G cells (1 × 10^6^/mL) were incubated in sDMEM containing 20 mM HEPES in the presence or absence of 40 mM 2-DG. (**A**,**B**) Effect of oligomycin on CCCP-induced _max_OCR (**A**) and estimated SRC (**B**) for T98G cells. The experiments were conducted by adding DMSO (0.5 µL) or 1 µg/mL oligomycin (Oligo) after a stable basal respiration rate was reached, followed by sequential additions of CCCP (2 µM each). Statistically significant difference in relation to the control (DMSO), **P < 0.01. (**C**) Measurements of ATP levels in T98G cells. Data were normalized as a percentage of the values of ATP in DMSO in the absence of 2-DG (% DMSO-Glucose). Statistically significant difference in relation to the control (DMSO), **P < 0.01.

**Figure 8:**
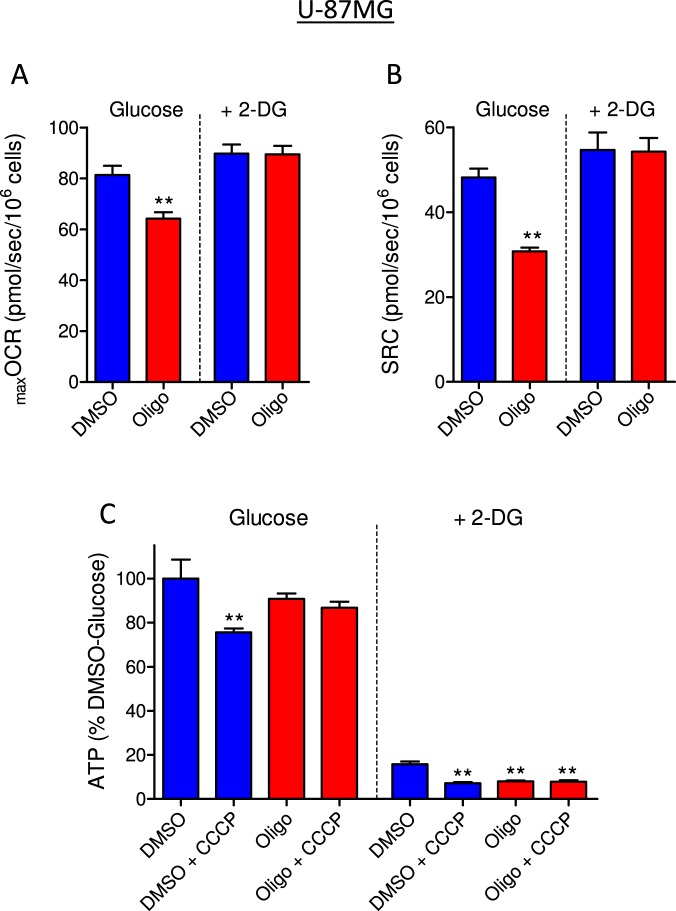
Effect of 2-DG on oligomycin-induced underestimation of _max_OCR and on ATP levels in U-87MG glioma cells. The experiments were conducted with U-87MG cells (2 × 10^6^/mL) under the same conditions described in Fig. 7 for T98G cells. Statistically significant difference in relation to the control (DMSO), **P < 0.01.

As we reported previously^14^, supplementing the medium with pyruvate only slightly decreased the oligomycin-induced underestimation of _max_OCR and SRC, suggesting that this effect is not associated with a limited supply of respiratory substrates to mitochondria. We hypothesized that this effect might be associated with the high glycolytic activity of tumor cells. To investigate the role of glycolysis in the underestimation of _max_OCR and SRC in the presence of oligomycin, the influence of this metabolic pathway was minimized in two different ways. First, cells were incubated in DMEM without glucose and pyruvate but containing 4 mM glutamine, a substrate that is metabolized to produce α-ketoglutarate, an intermediate of the citric acid cycle. For comparison, experiments were also conducted using DMEM containing all metabolic substrates (11 mM glucose, 1.25 mM pyruvate and 4 mM glutamine) (Figs 5 and 6). Second, cells were incubated in supplemented DMEM (sDMEM) containing the glycolytic inhibitor 2-deoxyglucose (2-DG; 40 mM), a glucose analog metabolized by hexokinase at the expense of ATP, generating the non-metabolizable molecule 2-deoxyglucose-6-phosphate and thus partially inhibiting glycolysis^16,17^ (Figs 7 and 8). The use of these two approaches to investigate the role of glycolysis is important since 2-DG also has effects on signal transduction that are unrelated to glycolysis inhibition^18^.

Figures 5 and 6 show that using only glutamine as the metabolic substrate, the underestimation caused by oligomycin on _max_OCR (Figs 5C and 6A) and SRC (Figs 5D and 6B) was not observed in either T98G or U-87MG cells. Measurements of OCR in T98G cells showed a higher basal OCR with DMEM containing only glutamine than with DMEM containing all the substrates (Fig. 5A,B), an effect likely related to the absence of the glycolytic metabolism-induced inhibition of oxidative phosphorylation (i.e., the Crabtree effect). Notably, SRC in the presence of glutamine alone was lower than in the presence of all the substrates under DMSO conditions. This decrease in SRC was expected because basal respiration increases with glutamine but _max_OCR does not. The data in Fig. 5E indicate that in medium containing only glutamine, oxidative metabolism in T98G cells cannot maintain the cellular ATP levels observed under control conditions (all substrates). As expected, inhibiting mitochondrial ATP synthesis with either CCCP or oligomycin decreased cellular ATP levels sustained by glutamine metabolism.

Figures 7 and 8 show that the glycolytic inhibitor 2-DG completely prevented the underestimation of _max_OCR (Figs 7A and 8A) and SRC (Figs 7B and 8B) due to oligomycin in T98G and U-87MG cells. These results suggest that underestimation of _max_OCR and SRC in the presence of oligomycin is associated with high cellular glycolytic activity, a predominant pathway for ATP regeneration in these tumor cells^19^. Next, cellular ATP content was measured under the same conditions used in the respirometry experiments, i.e., both cell lines were incubated in the presence or absence of oligomycin in sDMEM or sDMEM plus 40 mM 2-DG.

**Figure 9:**
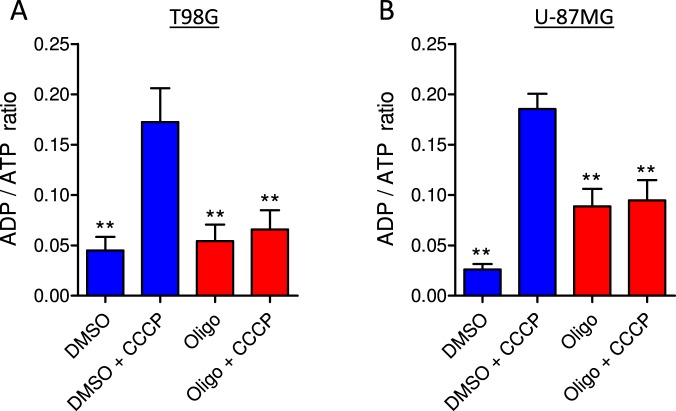
Determination of ADP/ATP ratio in T98G and U-87MG glioma cells. Cells were incubated in sDMEM containing 20 mM HEPES with and without oligomycin (in DMSO) and with and without CCCP as indicated. Statistically significant difference in relation to the condition “DMSO + CCCP”, **P < 0.01.

When ATP synthase was inhibited using oligomycin, no significant drop in ATP levels was observed in T98G and U-87MG cells incubated with sDMEM (Figs 7C and 8C), indicating that the ATP measured is produced mainly by glycolysis. When cells were incubated in sDMEM plus CCCP, ATP levels decreased by 26.6 ± 6.6% in T98G cells and 21.6 ± 6.1% in U-87MG cells in relation to the control (DMSO). Under this condition, mitochondrial ATP synthase is expected to hydrolyze ATP to restore the ∆Ψ_m_ that the protonophore CCCP dissipated^20,21^. In fact, when CCCP was added in the presence of oligomycin, cellular ATP levels were maintained because ATP synthase was prevented from operating in the reverse mode. In addition, the results in Fig. 9 show that the decrease in ATP levels induced by CCCP was accompanied by increased ADP/ATP ratios in both T98G and U-87MG cells in a mechanism sensitive to oligomycin.

Incubating the cells in sDMEM containing 2-DG resulted in a significant drop in ATP levels of 75.6 ± 4.0% in T98G cells and 83.5 ± 1.9% in U-87MG cells (Figs 7C and 8C), further suggesting that most cellular ATP in glioma cell lines is produced by glycolysis^19^. Induction of the Crabtree effect by 2-DG^22^ may also contribute to ATP depletion under this condition. When CCCP, oligomycin or oligomycin plus CCCP were present with 2-DG, the ATP levels in T98G cells dropped by 91.0 ± 1.2%, 86.0 ± 1.7% and 86.8 ± 1.8%, respectively (Fig. 7C). The same pattern was observed when U-87MG cells were tested with 2-DG (Fig. 8C).

**Figure 10:**
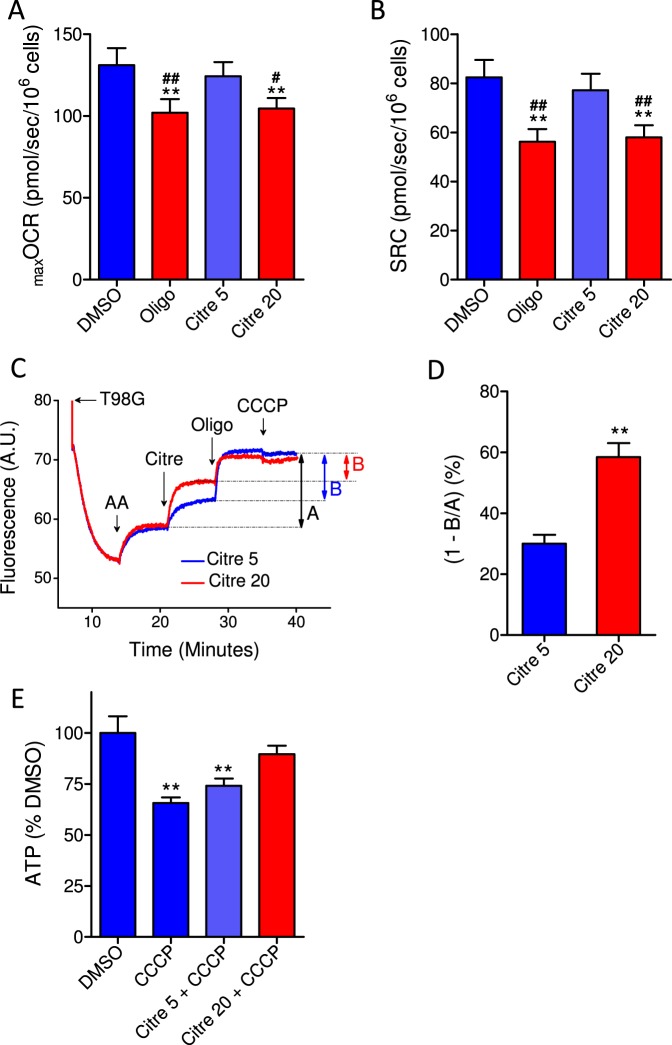
Effect of citreoviridin on CCCP-induced _max_OCR and ATP levels in T98G glioma cells: correlation with its inhibitory effect on the reverse activity of ATP synthase. T98G cells (1.5 × 10^6^/mL) were incubated in sDMEM containing 20 mM HEPES for OCR measurements, and the same medium without phenol red was used to estimate mitochondrial membrane potential. (**A**,**B**) Effect of 5 µM and 20 µM citreoviridin on CCCP-induced _max_OCR (**A**) and estimated SRC (**B**) in T98G cells. The experiments were conducted by adding DMSO (0.5 µL), 1 µg/mL oligomycin (Oligo), 5 µM citreoviridin or 20 µM citreoviridin after a stable basal respiration rate was reached, followed by sequential additions of CCCP (2 µM each) to obtain _max_OCR. Statistically significant difference in relation to the control (DMSO), **P < 0.01. Statistically significant difference in relation to treatment with 5 µM citreoviridin (citre 5), ^#^*P* < 0.05 and ^##^*P* < 0.01. (**C**) Representative traces of the citreoviridin effect on mitochondrial membrane potential sustained by the reverse activity of ATP synthase. Cells were incubated in sDMEM without phenol red and with 500 nM TMRM and 1 µM TPB^−^. Where indicated by the arrow, 1 µM antimycin (AA) was added, followed by addition of 5 µM or 20 µM citreoviridin (Citre). Next, 1 µg/mL oligomycin was added to completely inhibit the reverse activity of ATP synthase, followed by addition of 5 µM CCCP. The fraction of mitochondrial membrane potential that is maintained by the reverse activity of ATP synthase is indicated by the letter “A”, while the fraction that is insensitive to citreoviridin is indicated by the letter “B”. (**D**) Estimation of the membrane potential fractions that are maintained by the reverse activity of ATP synthase and are sensitive to 5 µM and 20 µM citreoviridin (1 - B/A). Statistically significant difference in relation to 5 µM citreoviridin (Citre 5), **P < 0.01. (**E**) Effect of CCCP on ATP levels of T98G cells incubated with 5 µM or 20 µM citreoviridin. Data were normalized as a percentage of the values of ATP in DMSO (% DMSO). Statistically significant difference in relation to the control (DMSO), **P < 0.01.

To investigate the importance of the inhibitory effect of oligomycin on the reverse activity of ATP synthase, we conducted experiments with citreoviridin. Low concentrations of citreoviridin inhibit the forward reaction of ATP synthase, with a minor effect on the reverse activity of this enzyme. However, higher concentrations of citreoviridin can inhibit forward and reverse activities^23,24^. Two different concentrations of citreoviridin were tested: 5 µM and 20 µM. Figure 10(A,B) shows that 5 µM citreoviridin did not affect _max_OCR and SRC. However, as shown previously^14^, in the presence of 20 µM citreoviridin, _max_OCR and SRC were underestimated by 18.9 ± 4.5% and 26.7 ± 5.9%, respectively, an effect similar to that observed in the presence of oligomycin.

The inhibitory effect of citreoviridin on the forward activity of ATP synthase was assessed based on its effect on basal OCR and compared with the effect of 1 µg/mL oligomycin^14^. The inhibitory effect on the reverse activity of ATP synthase was estimated by measuring the dissipation of ∆Ψ_m_ under conditions in which the respiratory chain was inhibited by antimycin A (Fig. 10C). In our previous study, the forward activity of ATP synthase, which was measured indirectly as the fraction of OCR due to ATP turnover, was inhibited by 84.8 ± 1.7% in the presence of 5 µM citreoviridin and was completely inhibited by 20 µM citreoviridin (see Fig. 5A,B in^14^). However, the membrane potential maintained by the reversed activity of ATP synthase was only 30.0 ± 3.0% and 58.5 ± 4.7% sensitive to 5 µM and 20 µM citreoviridin, respectively (Fig. 10C,D). These results indicate that a low concentration (5 µM) of citreoviridin can almost completely inhibit the forward reaction of ATP synthase and has a small effect on its reverse activity. Interestingly, this low concentration of citreoviridin does not reflect the underestimation of _max_OCR and SRC produced by oligomycin.

Next, the effect of CCCP on ATP levels was measured in the presence of 5 and 20 µM citreoviridin (Fig. 10E). Compared with the control condition (DMSO), CCCP reduced ATP by 27.9 ± 6.9%. The lower concentration of citreoviridin (5 µM) did not significantly prevent the ATP drop induced by CCCP (ATP drop of 17.6 ± 8.6%). However, CCCP did not induce a significant drop in ATP levels in the presence of 20 µM citreoviridin. This latter result is in accordance with the important inhibitory effect of citreoviridin at a high concentration (20 µM) on the reverse activity of ATP synthase.

### ATP, but not ADP, inhibits _max_OCR in digitonin-permeabilized T98G and U-87MG glioma cells

**Figure 11:**
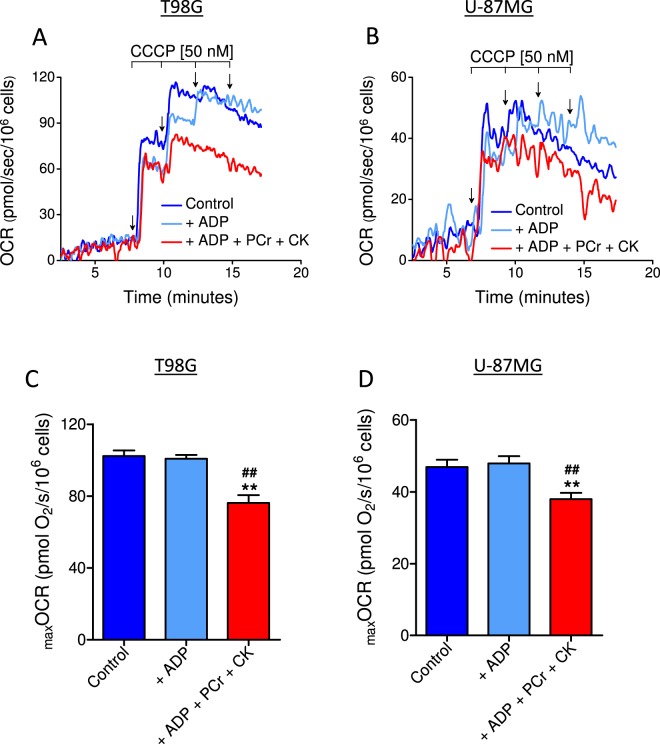
Effect of ADP and ATP on CCCP-induced _max_OCR in permeabilized T98G and U-87MG cells. T98G (1.5 × 10^6^/mL) and U-87MG (2 × 10^6^/mL) cells were incubated in “permeabilization medium” containing 30 µM digitonin, and _max_OCR was estimated by sequential additions of CCCP (0.05 µM each). The effects of ADP and ATP on _max_OCR were assessed by incubating the cells in the presence of 1 mM ADP or an ATP regeneration system (1 mM ADP, 10 mM phosphocreatine plus 50 µg/mL creatine phosphokinase). (**A**,**B)** Representative traces of OCR in permeabilized T98G and U87-MG cells. (**C**,**D)** Effects of ADP and ATP on CCCP-induced _max_OCR in permeabilized T98G and U87-MG cells. Statistically significant difference versus the corresponding control, **P < 0.01. Statistically significant difference in relation to “ + ADP”, ^##^*P* < 0.01.

To investigate the direct influence of ATP on _max_OCR, digitonin-permeabilized cells were incubated in the presence of ADP or ATP (i.e., ADP plus phosphocreatine and creatine phosphokinase, an ATP regeneration system) (Fig. 11). Permeabilized cells were used to allow easy adjustment of extramitochondrial ADP and ATP levels and to assess their effects on CCCP-induced _max_OCR. The presence of only ADP did not inhibit _max_OCR in either cell line; however, the presence of ATP inhibited _max_OCR by 26.0 ± 2.7% in T98G cells and 19.3 ± 1.8% in U-87MG cells.

### Effects of oligomycin and CCCP on mitochondrial membrane potential (∆Ψm) and OCR in human glioma cell lines

**Figure 12:**
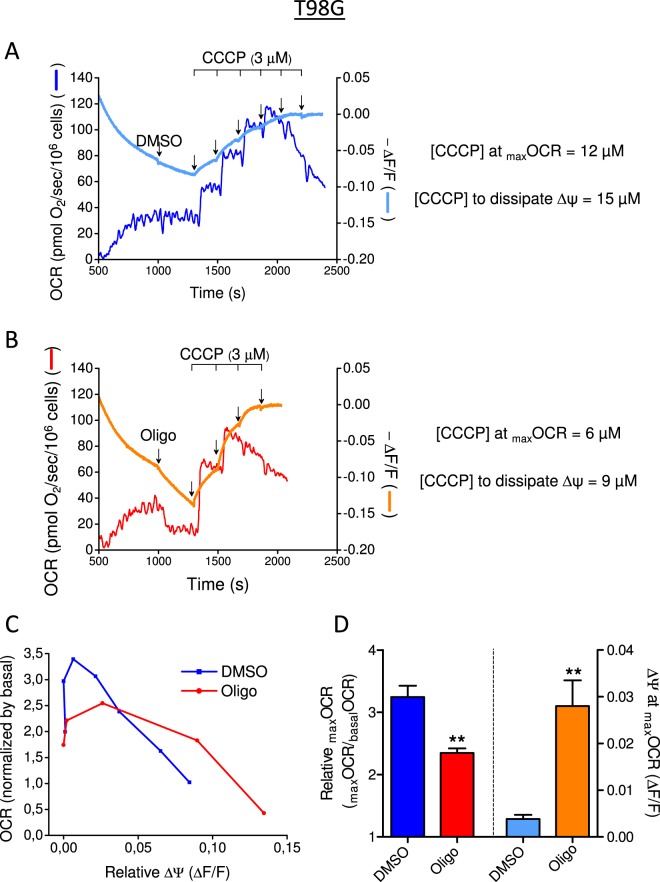
Monitoring mitochondrial OCR and membrane potential in T98G human glioma cells: effect of oligomycin on CCCP-induced _max_OCR. T98G cells (1.5 × 10^6^/mL) were resuspended in sDMEM without phenol red containing 20 mM HEPES, 500 nM TMRM and 1 µM TPB^−^. (**A**,**B**) Representative traces of mitochondrial OCR and membrane potential in suspended T98G cells. Where indicated by arrows, DMSO (0.5 µL) or 1 µg/mL oligomycin (Oligo) was added, followed by sequential additions of CCCP (3 µM each). OCR is shown on the left ordinate axis, and the mitochondrial membrane potential on the right axis. Membrane potential is expressed as −∆F/F, where F is the fluorescence intensity after the last addition of CCCP and ∆F is F minus any given fluorescence intensity. (**C**) Graphical correlation of mitochondrial OCR and membrane potential. OCR values were normalized by the respective basal OCR. (**D**) The left ordinate axis shows the relative _max_OCR (_max_OCR/_basal_OCR) for T98G cells in the presence and absence of oligomycin. The right ordinate axis shows the mitochondrial membrane potential (∆F/F) when _max_OCR was achieved. Statistically significant difference in relation to the control (DMSO), **P < 0.01.

**Figure 13:**
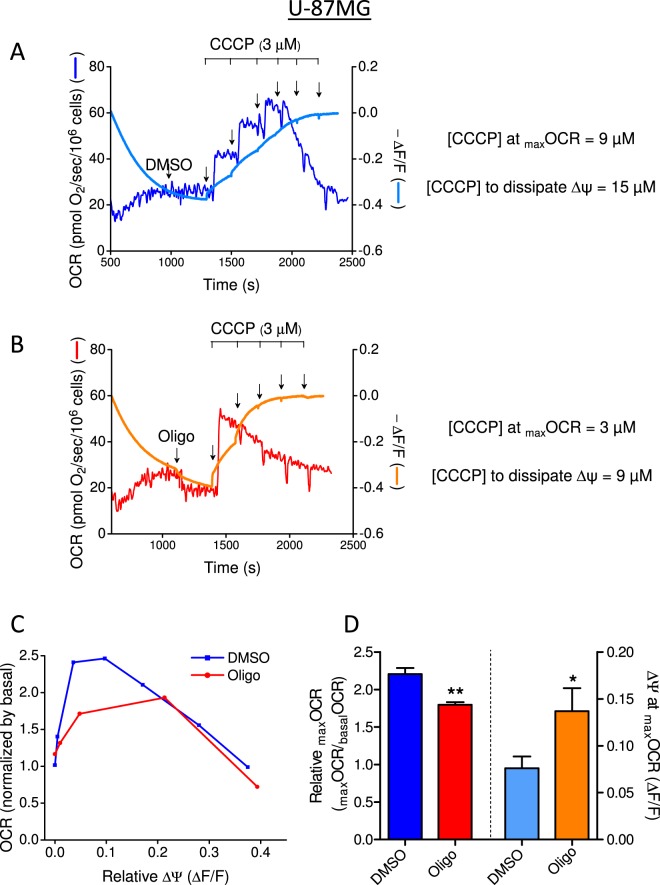
Monitoring mitochondrial OCR and membrane potential in U-87MG human glioma cells: effect of oligomycin on CCCP-induced _max_OCR. The experiments were conducted with U-87MG cells (2 × 10^6^/mL) using the same conditions described in Fig. 12 for T98G cells. Statistically significant difference in relation to the control (DMSO), *P < 0.05, **P < 0.01.

To further assess the underestimation of _max_OCR and SRC that occurs in the presence of oligomycin, OCR and mitochondrial membrane potential (∆Ψ_m_) were determined in parallel in intact T98G and U-87MG cells (Figs 12 and 13). A stable fluorescence signal of TMRM (500 nM) in sDMEM was obtained after approximately 8 min (not shown), after which cells were added. Oligomycin (or its vehicle DMSO) was then added to inhibit the activity of ATP synthase, and titration of the protonophore CCCP was carried out to progressively decrease ∆Ψ_m_ and achieve _max_OCR.

In DMSO without oligomycin, _max_OCR was reached with 12 µM and 9 µM CCCP for T98G and U-87MG cells, respectively (Figs 12A and 13A), whereas in the presence of oligomycin, lower CCCP concentrations (6 µM and 3 µM) were required (Figs 12B and 13B). However, higher protonophore concentrations were required to completely dissipate ∆Ψ_m_ in both cell lines: 15 µM in the absence of oligomycin and 9 µM in its presence. These results show that protonophore-induced _max_OCR occurred at a low ∆Ψ_m_, but complete dissipation of ∆Ψ_m_ led to inhibition of OCR (Figs 12C and 13C). This finding is in accordance with the inhibition of mitochondrial transport of substrates that can occur when ∆Ψ_m_ is dissipated^10,25–27^. Figures 12D and 13D show that _max_OCR was significantly underestimated in both cell lines when oligomycin was present and that _max_OCR was observed under higher ∆Ψ_m_ in the presence of oligomycin than in its absence (DMSO condition).

## Discussion

The results presented here indicate that the underestimation of CCCP-induced _max_OCR in tumor cell lines treated with oligomycin is caused by the high glycolytic activity of these cells. This conclusion is supported by the elimination of the inhibitory effect of oligomycin on _max_OCR when the glycolytic pathway was minimized using either glutamine as the only respiratory oxidative substrate (Figs 5 and 6) or the glycolytic inhibitor 2-DG (Figs 7 and 8). Furthermore, parallel determinations of OCR and ∆Ψ_m_ showed that _max_OCR was observed in the presence of a low ∆Ψ_m_ (Figs 12 and 13), which was higher when the estimation was performed in the presence of oligomycin (Figs 12D and 13D). This result indicates that _max_OCR cannot be reached in the presence of oligomycin because of limiting factors, as will be discussed later.

Measurements of cellular ATP levels and ADP/ATP ratios showed an association between the maintenance of intracellular ATP levels by glycolysis and the inhibitory effect of oligomycin on CCCP-induced _max_OCR (Figs 7–9**)**. When glycolysis was limited, either by using glutamine as the only metabolic substrate or by the presence of 2-DG, cellular ATP levels were not sustained. Under these conditions, a similar _max_OCR was obtained in both the presence and absence of oligomycin (Figs 5–8**)**. In the absence of this ATP synthase inhibitor, the decrease in glycolytic ATP in the presence of CCCP was mostly due to ATP consumption by the reverse activity of mitochondrial ATP synthase (Figs 5E, 7C and 8C). Even though slow reverse operation of ATP synthase is expected in respiration-impaired mitochondria with an intact inner membrane^28^, higher reverse activity occurs under conditions of strong uncoupling, e.g., in CCCP-induced _max_OCR or when the integrity of the inner mitochondrial membrane is disrupted.

The results of the experiments conducted with the ATP synthase inhibitor citreoviridin, a compound that affects the forward and reverse activity of ATP synthase differently^23,24^ (Fig. 10C,D), further support the proposition that cellular ATP levels are reduced by the reverse activity of ATP synthase under uncoupling conditions. A low concentration of citreoviridin (5 µM), which almost completely inhibited the forward activity of ATP synthase^14^ but had only a minor effect on the reverse activity (Fig. 10D), neither prevented a decrease in ATP levels by CCCP nor inhibited CCCP-induced _max_OCR. However, at a higher concentration (20 µM), citreoviridin inhibited the reverse activity of ATP synthase to a greater degree, preventing the drop in ATP caused by CCCP and resulting in the inhibition of CCCP-induced _max_OCR.

Exposing digitonin-permeabilized cells to exogenous ATP but not ADP resulted in partial inhibition of CCCP-induced _max_OCR (Fig. 11). Maintenance of a higher intracellular ATP/ADP ratio may limit CCCP-induced _max_OCR by inhibiting enzymes involved in the reduction of NAD^+^ to NADH in the mitochondrial matrix, thereby restricting electron transfer from carbon substrates to ETS. According to previous studies, a higher ATP/ADP ratio in the mitochondrial matrix is associated with lower activity of pyruvate dehydrogenase (PDH), isocitrate dehydrogenase-3 (IDH-3) and glutamate dehydrogenase (GDH). ADP stimulates PDH activity by inhibiting pyruvate dehydrogenase kinase, which phosphorylates and inactivates PDH^29,30^; a decrease in ATP/ADP ratio results in lower IDH-3 *K*_m_ values for its substrates^31,32^, and GDH is subject to positive allosteric regulation by ADP^33,34^. Interestingly, a recent study with astrocytes revealed that ADP-stimulated GDH plays an important role under conditions of increased mitochondrial oxidative metabolism demand^35^. This pathway may play an important role in the supply of mitochondrial NADH to glutamine-addicted highly glycolytic tumor cells^19,36^. In addition, a high intramitochondrial ATP/ADP ratio may also limit _max_OCR by promoting partial inhibition of NADH oxidation. Kadenbach’s group^37^ demonstrated that the binding of ADP and ATP to cytochrome *c* oxidase (respiratory complex IV) regulates the activity of this enzyme. Allosteric inhibition of cytochrome *c* oxidase by ATP occurs in the presence of a high intramitochondrial ATP/ADP ratio and may inhibit _max_OCR even in the presence of sufficient NADH^37,38^.

In the present study, ATP contents and ADP/ATP ratios were determined in total cell extracts. Because of the negative-inside mitochondrial membrane potential, the ADP/ATP ratio is significantly different between the cytosol and the mitochondrial matrix^39^. However, our main inferences are based on experimental conditions in which membrane potential is mostly dissipated (i.e., in the presence of CCCP or CCCP plus oligomycin). Under such conditions an equilibrium between the cytosol and mitochondrial matrix ADP/ATP ratios is expected^39^.

Significant inhibition of protonophore-induced _max_OCR was also observed by Kim *et al*.^40^ in an oligomycin-treated INS-1E pancreatic beta cell line. This effect was associated with time-dependent exposure to oligomycin that was likely causing a progressive loss of cell function in the absence of oxidative phosphorylation^40^. However, our results with glioma cell lines seem to differ in nature from those observed in INS-1E cells because the limitation on _max_OCR was observed very soon after addition of oligomycin to cells sustaining high intracellular ATP levels. Given that many studies have evaluated mitochondrial oxidative metabolism in highly proliferative tumor cell lines (i.e., highly glycolytic cells), the mechanism of oligomycin-mediated underestimation of _max_OCR reported here is likely to be more widespread in experimental protocols.

We conclude that high glycolytic activity leads to the underestimation of CCCP-induced _max_OCR and SRC in tumor cells treated with oligomycin. Under these conditions, oligomycin maintains cellular ATP levels by preventing the reverse activity of ATP synthase. Minimizing glycolytic activity may allow more accurate assessment of _max_OCR in the presence of ATP synthase inhibitors.

## Methods

### Chemicals

Adenosine diphosphate (ADP; catalog number: A2754), carbonyl cyanide 3-chlorophenyl hydrazone (CCCP; cat. C2759), creatine phosphokinase from rabbit muscle (CK; cat. C3755), 2-deoxy-D-glucose (2-DG; cat. D8375), digitonin (cat. D141), dimethylsulfoxide (DMSO; cat. D8418), L-glutamic acid (cat. G1251), L-glutamine (cat. G3126), L-malic acid (cat. M1000), nucleoside 5′-diphosphate kinase from *S*. *cerevisiae* (NDK; cat. N0379), oligomycin (oligo; cat. O4876), phosphocreatine disodium salt (PCr; cat. P7936), pyruvic acid sodium salt (cat. P4562) and sodium tetraphenylboron (TPB^−^; cat. T4125) were purchased from Sigma-Aldrich (St Louis, MO, USA). The oligomycin compound is a mixture of three oligomycins (A, B and C, where oligomycin A represents approximately 65% of the mixture). Deoxycytidine triphosphate (dCTP) solution (cat. 10217–016) and tetramethylrhodamine methyl ester (TMRM; cat. T668) were supplied by Thermo Fisher Scientific (Waltham, MA, USA) and citreoviridin (citre; cat. 11319) by Cayman Chemical (Ann Arbor, MI, USA).

CCCP, citreoviridin, oligomycin and TMRM stock solutions were prepared in DMSO; CK, 2-DG, glutamate, glutamine, HEPES, malate, NDK, PCr, pyruvate and TPB^−^ stock solutions were prepared in deionized water; ADP and HEPES solutions were adjusted to pH 7.2 with NaOH; and glutamate, malate and pyruvate solutions were adjusted to pH 7.2 with KOH.

Dulbecco’s modified Eagle’s medium (DMEM), with or without 5.5 or 11 mM glucose, 1.25 mM pyruvate, 4 mM glutamine, 44 mM sodium bicarbonate and 15 mg/L phenol red, were all supplied by Vitrocell (Campinas, São Paulo, Brazil). Antibiotics (1  ×  10^4^ U/mL penicillin plus 10 mg/mL streptomycin) and fetal bovine serum (FBS) were also supplied by Vitrocell. Unless otherwise specified, DMEM contained 11 mM glucose, 4 mM glutamine, 1.25 mM pyruvate, 44 mM sodium bicarbonate and 15 mg/L phenol red. The concentrations of the energy substrates are within the ranges normally used in cultures and cell metabolic analysis^12,13^.

### Cell Lines and Cell Culture

The human glioblastoma T98G and U-87MG cell lines were purchased from the American Type Culture Collection (Manassas, VA, USA) and cultured as previously described^14^. On the day of the experiment, the cells were trypsinized and resuspended (16–32 × 10^6^ cells/mL; >95% viability) in DMEM supplemented with 10% FBS, 100 U/mL penicillin and 100 µg/mL streptomycin (sDMEM) containing 20 mM HEPES. For experiments testing the components of the medium (glucose 5.5 mM or 11 mM, glutamine, sodium bicarbonate and FBS), cells were resuspended in the corresponding experimental medium as described in the figure legends. Cell suspensions were maintained at room temperature (ca. 23 °C) and used within 2.5 h.

The data reported here are from experiments conducted over 18 months; as the cells were expanded from different frozen aliquots, and the components of the medium (e.g., FBS) were from different batches, absolute mean values of some variables can be expected to vary (e.g., _max_OCR can oscillate up to 25%) more than the standard error of the mean when experiments performed a couple of months apart are compared. Nonetheless, we would emphasize that each experimental protocol was conducted within 2 to 4 weeks and that these variations were not observed; moreover, the effects of treatments were consistent throughout the whole study.

### Measurement of OCR in suspended tumor cells

The OCR in intact and permeabilized suspended cells was determined using a respirometer (OROBOROS Oxygraph-2k, Innsbruck, Austria), as previously described^14,19^. In intact tumor cells this was carried out by incubating an aliquot of the cell suspension (2–4 × 10^6^ cells) at 37 °C in a 2 mL chamber containing the reaction medium, as described in the figure legends. OCR was measured in permeabilized cells by incubating 3–4 × 10^6^ cells at 37 °C in 2 mL of “permeabilization medium” containing 125 mM sucrose, 65 mM KCl, 2 mM K_2_HPO_4_, 1 mM MgCl_2_, 1 mM EGTA, 1 µg/mL oligomycin, 10 mM HEPES-K^+^ pH 7.2 and respiratory substrates (1 mM glutamate, 0.5 mM malate and 1 mM pyruvate), as well as digitonin (30 µM) for plasma membrane permeabilization. The concentrations of the respiratory substrates in the “permeabilization medium” were chosen to better resemble those found *in situ* (submillimolar levels), enabling the inhibitory effect of adenine nucleotides on the oxidative metabolism of these substrates to be studied.

### Measurement of ATP content and ADP/ATP ratio in suspended tumor cells

T98G cells (1.5 × 10^6^/mL) and U-87MG cells (2 × 10^6^/mL) were resuspended in DMEM or sDMEM containing 20 mM HEPES. DMSO or oligomycin (1 µg/mL) was added to the cell suspension samples and incubated for 4–5 min, and CCCP (1 µM for DMEM or 10 µM for sDMEM) was then added to some of the samples and incubated for an additional 10–12 min. Cell suspensions were immediately centrifuged (6,000 *g*, 5 min), the supernatants were discarded, and the pellets were homogenized in 1 mL of lysis buffer (25 mM TRIS-phosphate, pH 7.8, 2 mM dithiothreitol, 2 mM EDTA, 10% glycerol and 1% Triton X-100) compatible with the luciferase assay reagents and maintained for 5 min in an ice bath. Control experiments showed that the centrifugation step did not significantly impair cell viability (results not shown). Samples were then analyzed for ATP content using a luminescence assay (CellTiter-Glo Luminescent Cell Viability Assay, Promega, G7570) in a plate reader (SpectraMax M3 – Molecular Devices, Sunnyvale, CA, USA). Luminescence was read at 560 nm in endpoint mode with an integration time of 1 sec. SoftMax Pro 6.4 software was used for data acquisition. To estimate ADP content, 100 µM dCTP and 10 U/mL NDK were added and luminescence was read again after 10 min^41^.

### Mitochondrial membrane potential measurements in suspended intact tumor cells

The mitochondrial membrane potential in suspended intact cells was evaluated with the fluorescent probe TMRM on a Hitachi F7000 spectrofluorometer (Tokyo, Japan) equipped with magnetic stirring and operating with excitation and emission wavelengths of 553 and 576 nm, respectively, and a response time of 2 sec. Slit width was 2.5 nm for excitation and 5.0 nm for emission. Cell suspensions were trypsinized and resuspended in phenol red-free sDMEM containing 20 mM HEPES, and 2–4 × 10^6^/2 mL cells were added to sDMEM containing 20 mM HEPES, 500 nM TMRM and 1 µM TPB, which was present to facilitate TMRM equilibrium across the plasma membrane^42^. Simultaneous measurements of OCR were taken in the chamber of the OROBOROS respirometer under identical experimental conditions.

Membrane potential, expressed as ∆F/F, was calculated, where F is the fluorescence intensity after the last addition of CCCP (i.e., completely dissipated ∆Ψ_m_) and ∆F is F minus any given fluorescence intensity^43^.

### Statistical analysis

The results are shown as representative traces and/or the mean ± standard error of the mean (SEM). Experiments were performed with cells from at least four different passages. Paired Student’s t-test was applied to analyze differences between two groups. Multiple comparisons were performed by repeated-measures one-way analysis of variance (ANOVA) and the *post hoc* Bonferroni test^14^.

## Data Availability

The datasets generated during the current study are available from the corresponding author on reasonable request.
